# Characterization of Regulatory B Cells in Graves’ Disease and Hashimoto’s Thyroiditis

**DOI:** 10.1371/journal.pone.0127949

**Published:** 2015-05-27

**Authors:** Birte Kristensen, Laszlo Hegedüs, Steven K. Lundy, Marie K. Brimnes, Terry J. Smith, Claus H. Nielsen

**Affiliations:** 1 Institute for Inflammation Research, Department of Infectious Diseases and Rheumatology, Copenhagen University Hospital Rigshospitalet, Copenhagen, Denmark; 2 Department of Endocrinology and Metabolism, Odense University Hospital, Odense, Denmark; 3 Division of Rheumatology, Department of Internal Medicine, University of Michigan, Ann Arbor, MI, United States of America; 4 Department of Ophthalmology and Visual Sciences, Kellogg Eye Center and Division of Metabolism, Endocrinology, and Diabetes, Department of Internal Medicine, University of Michigan Medical School, Ann Arbor, MI, United States of America; COCHIN INSTITUTE, Institut National de la Santé et de la Recherche Médicale, FRANCE

## Abstract

A hallmark of regulatory B cells is IL-10 production, hence their designation as IL-10^+^ B cells. Little is known about the ability of self-antigens to induce IL-10^+^ B cells in Graves’ disease (GD), Hashimoto’s thyroiditis (HT), or other autoimmune disease. Here we pulsed purified B cells from 12 HT patients, 12 GD patients, and 12 healthy donors with the thyroid self-antigen, thyroglobulin (TG) and added the B cells back to the remaining peripheral blood mononuclear cells (PBMCs). This procedure induced IL-10^+^ B-cell differentiation in GD. A similar tendency was observed in healthy donors, but not in cells from patients with HT. In GD, B cells primed with TG induced IL-10-producing CD4^+^ T cells. To assess the maximal frequency of inducible IL-10^+^ B cells in the three donor groups PBMCs were stimulated with PMA/ionomycin. The resulting IL-10^+^ B-cell frequency was similar in the three groups and correlated with free T_3_ levels in GD patients. IL-10^+^ B cells from both patient groups displayed CD25 or TIM-1 more frequently than did those from healthy donors. B-cell expression of two surface marker combinations previously associated with regulatory B-cell functions, CD24^hi^CD38^hi^ and CD27^+^CD43^+^, did not differ between patients and healthy donors. In conclusion, our findings indicate that autoimmune thyroiditis is not associated with reduced frequency of IL-10^+^ B cells. These results do not rule out regulatory B-cell dysfunction, however. The observed phenotypic differences between IL-10^+^ B cells from patients and healthy donors are discussed.

## Introduction

Autoimmune thyroiditis (AITD) includes Graves’ disease (GD) and Hashimoto’s thyroiditis (HT), which are typically associated with hyper- and hypothyroidism, respectively. B cells are known to play an essential role in GD by virtue of their production of pathognomonic activating autoantibodies against the thyroid-stimulating hormone (TSH) receptor, leading to increased production and secretion of the thyroid hormones T_4_ and T_3_ and a compensatory decrease in TSH production by the anterior pituitary gland [1, 2]. It is unclear whether B cells also play a pathogenic role in HT. Autoantibodies to the thyroid self-antigens thyroglobulin (TG) and thyroid peroxidase (TPO) are commonly found in both GD and HT, but T-cell mediated destruction of thyroid architecture plays a central role in HT [3, 4]. This leads to low production of T_4_ and T_3_, and a compensatory increase in TSH production [3, 4]. The beneficial effect of the B cell-depleting antibody rituximab in a number of autoimmune diseases, including multiple sclerosis and type 1 diabetes mellitus, suggests a critical role for B cell endorsement in T-cell dominated diseases [5].

Recently, immunoregulatory B cells (Bregs) have been identified [6–8]. They contribute to maintenance of peripheral tolerance by virtue of their production of interleukin-10 (IL-10), transforming growth factor (TGF)-β, Fas ligand, and TRAIL expression [9]. Studies quantifying IL-10^+^ B cells have generally used polyclonal B-cell activation with toll-like receptor (TLR) agonists, phorbol-12-myristate-13-acetate (PMA), ionomycin, or anti-IgM/-IgG antibodies [10, 11]. While these approaches allow determination of immunoregulatory potential of circulating B-cells, they do not necessarily reflect the capacity of IL-10^+^ B cells to inhibit immune responses to specific self-antigens. Recently, we demonstrated that TG induces IL-10 production by a B-cell subset containing high proportions of CD5^+^ and CD24^hi^ cells [12].

Little is known about IL-10^+^ B-cell frequency or the ability of B cells to induce IL-10^+^ T cells in AITD. Here we investigated the capacity of B cells from patients with GD, HT, and those from healthy donors to differentiate into IL-10^+^ B cells when challenged with TG or the mitogen PMA/ionomycin. Moreover, we assessed the capacity of B cells pulsed with TG to induce IL-10 production by CD4^+^ T cells and cytokine release from intact peripheral blood mononuclear cells (PBMCs). Finally, the expression by IL-10^+^ B cells of several surface markers that have previously been associated with regulatory functions was examined.

## Methods

### Subjects

Whole blood from 12 healthy donors (demographics: 9 females, 3 males; median age 44 yrs) with no history of autoimmune disease was provided by the Blood Bank at Copenhagen University Hospital. A total of 12 patients with HT and 12 patients with GD, attending the Endocrinology outpatient clinic at Odense University Hospital between November 2013 and March 2014 participated in the study. HT patients were characterized by elevated serum TSH levels, raised serum TPO Ab and/or TG Ab levels, and undetectable anti-TSHR Abs. Suppressed serum TSH levels, increased free T_4_ (FT_4_) and free T_3_ (FT_3_) levels, elevated serum anti-TSHR Ab levels, diffuse uptake on thyroid scintigraphy, and ultrasound demonstrating diffuse hypoechogenicity typified those with GD.

All patients were diagnosed within three years of study participation with the exception of one HT patient diagnosed in 2009 and two GD patients diagnosed in 2008 and 2009. At the time of blood collection, 9 out of 12 GD patients were receiving methimazole (median: 10 mg/day, IQR: 5 – 15 mg/day) or levothyroxine (median: 75 μg/day, IQR: 50 – 150 μg/day), while 5 out of 12 HT patients were receiving levothyroxine (median: 112.5 μg/day, IQR: 100 – 125 μg/day). Duration of anti-thyroid treatment varied from 2 weeks to 5 years. Further clinical details of the study participants are outlined in Table 1. Written informed consent was obtained from all participating subjects prior to their participation. The study was approved by the Ethical Committee from the Region of Southern Denmark (project #28699) and followed the guidelines outlined in the Declaration of Helsinki.

**Table 1:** Patient characteristics

	Graves’ Disease (n = 12)	Hashimoto’s Thyroiditis (n = 12)
**Age (years)**		
*Median*	58	51
*Interquartile Range*	45.8 – 69.0	40.3 – 58.5
**Gender (% females)**	91.7	83.3
**Thyroid hormones levels**		
*TSH (mIU/L)* ^*1*^	0.7 (0.01 – 5.9)	5.4 (1.6 – 11.2)
* T*_*3*_ *(nmol/L))* ^*2*^	1.7 (1.1 – 3.6)	1.5 (1.4 – 1.6)
* T*_*4*_ *(nmol/L)* ^*3*^	77 (56.8 – 162.8)	78.0 (68.3 – 102.5)
* T*_*4*_ *uptake (nmol/L)* ^*4*^	0.9 (0.8 – 1.0)	1.0 (0.9 – 1.0)
* FT*_*3*_ ^*5*^	1.7 (1.2 – 4.0)	1.6 (1.4 – 1.7)
* FT*_*4*_ ^*6*^	82.4 (56.9 – 179.1)	79.7 (71.7 – 109.3)
**Autoantibodies**		
* Anti-TSHR (kIU/L)* ^*7*^	9.4 (3.6 – 14.7)	Negative
* Anti-TPO (kIU/L)* ^*8*^	228.5 (117.8 – 958.0)	641.5 (285 – 820.5)

All thyroid hormone and autoantibody levels are taken at the time of immunological studies. Data are displayed as median and IQR.

^1^Normal range: 0.3 – 4.0 mIU/L

^*2*^Normal range: 1.3 – 2.2 nmol/L

^3^Normal Range: 60 – 130 nmol/L

^4^Normal Range: 0.6 – 1.2 nmol/L

^5^FT_3_ or FT_4_ were defined as T_3_ or T_4_ divided by T_4_ uptake

^6^FT_3_ or FT_4_ were defined as T_3_ or T_4_ divided by T_4_ uptake

^7^Positive >1.0 kIU/L

^8^Normal range is 2.1 – 9.8 kIU/L [13, 14]

TSH = thyroid stimulating hormone; TSHR = thyroid stimulating hormone receptor; TPO = thyroid perioxdiase.

### Biochemistry

Serum TSH was measured using the Immulite 2000 assay (Siemens, Erlangen, Germany). T_4_ and T_3_ were measured by time-resolved fluoroimmunoassays (TRFIA) using an AutoDELFIA instrument (Perkin Elmer/Wallac, Turku, Finland). FT3 and FT_4_ were defined as T_3_ or T_4_ divided by T_4_ uptake. Anti-TPO Abs and anti-TG Abs were measured by TRIFA, and anti-TSHR Abs were measured by DYNOtest TRAK human radio receptor assay (Brahms Diagnostica, Berlin, Germany).

Reference ranges: TSH 0.3 – 4.0 mIU/L; T_3_ 1.3 – 2.2 nmol/L; T_4_ 60 – 130 nmol/L; T_4_ uptake 0.6 – 1.2 nmol/L; anti-TPO Abs 2.1 – 9.8 kIU/L; and anti-TG Abs 2.9–19.2 kIU/L. Samples were regarded as negative for anti-TSHR Abs < 0.7 kIU/L [13, 14].

### Cells and Serum

PBMCs were isolated by density gradient separation using LymphoPrep (cat #1114547; Axis-Shield, Oslo, Norway) at 1200 x *g* for 30 minutes, washed twice in phosphate buffered saline followed by centrifugation at 400 x *g*, and re-suspended in RPMI 1640 (cat #01-106-1a; Biological Industries, Kibbutz Beit Haemek, Israel) containing 30% (v/v) AB serum, 25 mM HEPES, 2 mM L-glutamine (cat #25030–024; Gibco, Life Technologies, Carlsbad, CA), and 50 μg/mL gentamycin sulfate (cat #03-035-1c; Biological Industries). All subsequent centrifugations were carried out at 400 x *g*. Human serum isolated from healthy male donors of blood group AB (AB serum) was purchased from Lonza (cat # 14-490E; Basel, Switzerland) and used as the serum source in all experiments.

### B-cell purification

B cells were positively selected from PBMCs using the human CD19 Positive Cell Isolation kit (cat #18054; EASY SEP, StemCell Technologies, Vancouver, Canada). In brief, PBMCs were re-suspended at 1x10^8^ cells/mL and incubated at 4^°^C with positive selection cocktail (100 μl/mL) for 15 minutes and magnetic nanoparticles (50 μL/mL) for 10 minutes. PBMCs underwent 4 x 5 minutes separations to increase purity which was assessed by staining with anti-CD19 APC (cat #555–415; BD Bioscience, San Jose, CA), anti-CD3 PerCP (cat #552851; BD Bioscience), and anti-CD14 FITC (cat #555–397; BD Bioscience).

### Stimulation of cell cultures

Polyclonal stimulation of B cells was conducted using CpG oligodeoxynucleotides (ODN) 2006 (10 μg/mL; cat #tlrl-2006-1; InvivoGen, San Diego CA) or a cell stimulation cocktail containing phorbol 12-myristate 13-acetate (PMA) and ionomycin (cat #004970–91; eBiosicence, San Diego, CA). The final working concentration of PMA was 50 ng/mL and 1 μg/mL for ionomycin. For PMA/ionomycin stimulation, 1x10^6^ PBMCs were cultured in RPMI 1640 media with 30% (v/v) pooled serum from blood group AB-positive donors and 2 μL PMA/ionomycin cell stimulation cocktail was added to each well for 4 h at 37^°^C. In other studies, B cells were stimulated with TG (30 μg/ml, MW 660 kDa) purified from human thyroid tissue (cat #OPSA10707; Aviva Systems Biology, San Diego, CA). Limulus amebocyte lysate (cat #50-647U; QCL -1000 Chromogenic LAL, Lonza) assay revealed endotoxin, which was removed using Triton X-114 as previously described [15].

### Pulsing of B cells with antigen

Purified B cells were either preloaded with TG (30 μg/mL) or CpG ODN (10 μg/mL) for 1 h at 37^°^C or received no antigen and served as the negative control. Unless otherwise stated, 1.0x10^5^ B cells were co-cultured with 2.0x10^5^ residual PBMCs in RPMI 1640 media containing 30% (v/v) AB serum for 48 h. Supernatants were collected after 48 h of incubation and analyzed for IL-10 and IL-6 using Luminex (Austin, TX). Multiplex beads were supplied by BioRad (Hercules, CA).

### Intracellular staining for IL-10

Cells were fixed and permeabilized using CytoFix/CytoPerm (cat #554–722; BD Biosciences). IL-10 staining was assessed, following stimulation with PMA/ionomycin, TG, or CpG (4 and 48 h, respectively). Brefeldin A (cat #420601; Biolegend, San Diego, CA) was added (1 μL/well) to PBMCs at the beginning of stimulation. For 48 h stimulations, Brefeldin A was added for the final 5 h.

### Flow cytometry

After 4 h of stimulation, PBMCs were stained with a combination of the following antibodies from BD Bioscience: anti-CD19-PerCP (cat #345–778), anti-CD43-FITC (cat #555–475), anti-CD27-PECy7 (cat #560–609), anti-CD24-FITC (cat #555–427), anti-CD38-PECy7 (cat #335–825), anti-CD25 FITC (cat #555–431), anti-IL-10 APC (cat #554–707) or anti-IL-10-PE (cat #559–330). Anti-CD5 APC was from Dako (cat #C7242, Glostrup, Denmark) and anti-TIM-1 PE was purchased from Biolegend (cat #353–904).

After 48 h of stimulation, IL-10 was detected with the following BD Bioscience Abs: anti-CD19 APC (cat #555–415), anti-CD14 FITC (cat #555–397), anti-CD4 PerCP (cat #345–770), anti-CD8 PECy7 (cat #557–746) and anti-IL-10 PE (cat #559–330). Live/Dead Fixable Near InfraRed staining (cat #L10119; Molecular Probes, Invitrogen, Carlsbad, CA) was included.

The cells were acquired with a FACS Canto (BD Bioscience) flow cytometer with argon laser (488 nm) and Helium-Neon laser (633nm) excitation.

All analyses were carried out using FlowJo V10 (TreeStar, Ashland, OR). Dead cells were excluded based on Live/Dead Fixable Near InfraRed staining and B cells were identified as CD19^+^ events within a morphological lymphocyte gate. Individual IL-10^+^ B cells were identified using the gating strategy demonstrated in S1 Fig.

### Statistics

Comparisons between each patient group and healthy donors were performed using the two-tailed Mann-Whitney U-test. Differences between HT and GD patients were considered subordinate. In addition, comparisons within each group were performed using the Wilcoxon matched-pairs signed rank test. Correlations between thyroid hormones (FT_3_, FT_4_ and TSH) and IL-10^+^ B cell frequency were evaluated using Spearman Rank correlation coefficient. All analyses were carried out using GraphPad Prism version 6 (GraphPad Software, La Jolla, CA). P values are presented in the figures. P-values < 0.05 were considered significant.

## Results

### Induction of IL-10^+^ B cells by the thyroid self-antigen TG

**Fig 1:**
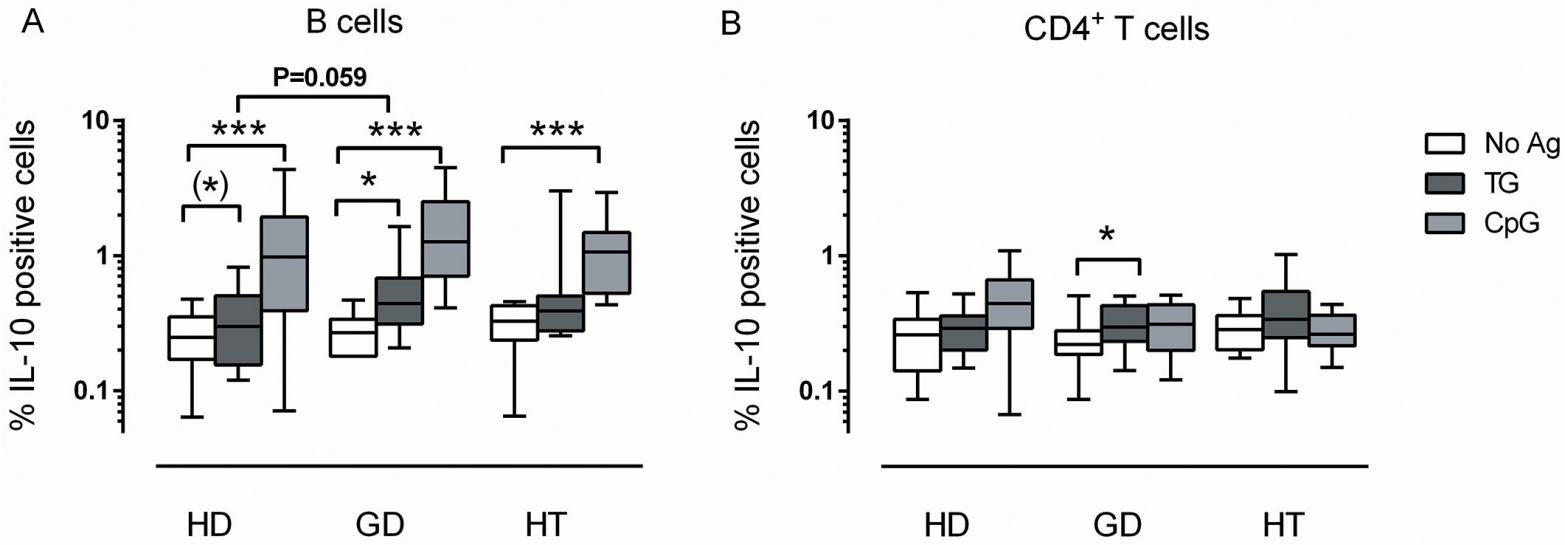
Induction of IL-10^+^ B cells and CD4^+^ T cells by the self-antigen thyroglobulin. CD19^**+**^ B cells were purified from PMBCs from healthy donors (HD; N = 12), patients with Graves’ disease (GD; N = 12), or patients with Hashimoto’s thyroiditis (HT; N = 12). 1x10^**5**^ purified B cells were pulsed with thyroglobulin (TG), a positive control stimulus, CpG oligodeoxynucleotide (CpG), or no antigen (No Ag) for 1 h, and co-cultured with 2x10^**5**^ of the remaining PBMCs for 48 hours before staining for (A) CD19^**+**^ B cells, (B) CD4^**+**^ T cells, and for intracellular IL-10, followed by flow cytometric analysis. Brackets show differences between groups with the corresponding raw P-values (Mann-Whitney U test). ^(^*^)^ P = 0.10; * P<0.05; *** P<0.001 within groups (Wilcoxon matched-pairs signed rank test).

In general, polyclonal stimulation with PMA/ionomycin or CpG has been used to study cytokine production by B cells. While this approach may show the potential of the entire B cell pool to differentiate into cytokine-producing cells, it does not reflect the more physiological situation where B cells may be stimulated clonally with self-antigens and receive help from antigen-specific T-helper cells. To mimic such conditions, B cells were purified and pulsed with the thyroid self-antigen TG before they were added back to the remaining PBMCs (Fig 1).

The hallmark of immunoregulatory function in B cells is the production of IL-10 [9]. As shown in Fig 1A, exposure to TG significantly increased the proportion of IL-10^+^ B cells in cultures from GD patients (p = 0.01). A similar tendency was observed among healthy donors (p = 0.10), but not among HT patients. After pulsing with TG, cultures derived from GD patients tended to contain more IL-10^+^ B cells than cultures derived from healthy donors (p = 0.059). As expected, CpG induced IL-10^+^ B cells substantially in all three donor groups.

We next examined whether uptake and presentation of TG by B cells promoted induction of IL-10 producing T cells in the co-cultures. As shown in Fig 1B, pulsing of B cells with TG resulted in an increase in the proportion of IL-10-producing CD4^+^ T cells in cultures derived from GD patients (p = 0.01), but not in cultures from HT patients or healthy donors.

**Fig 2:**
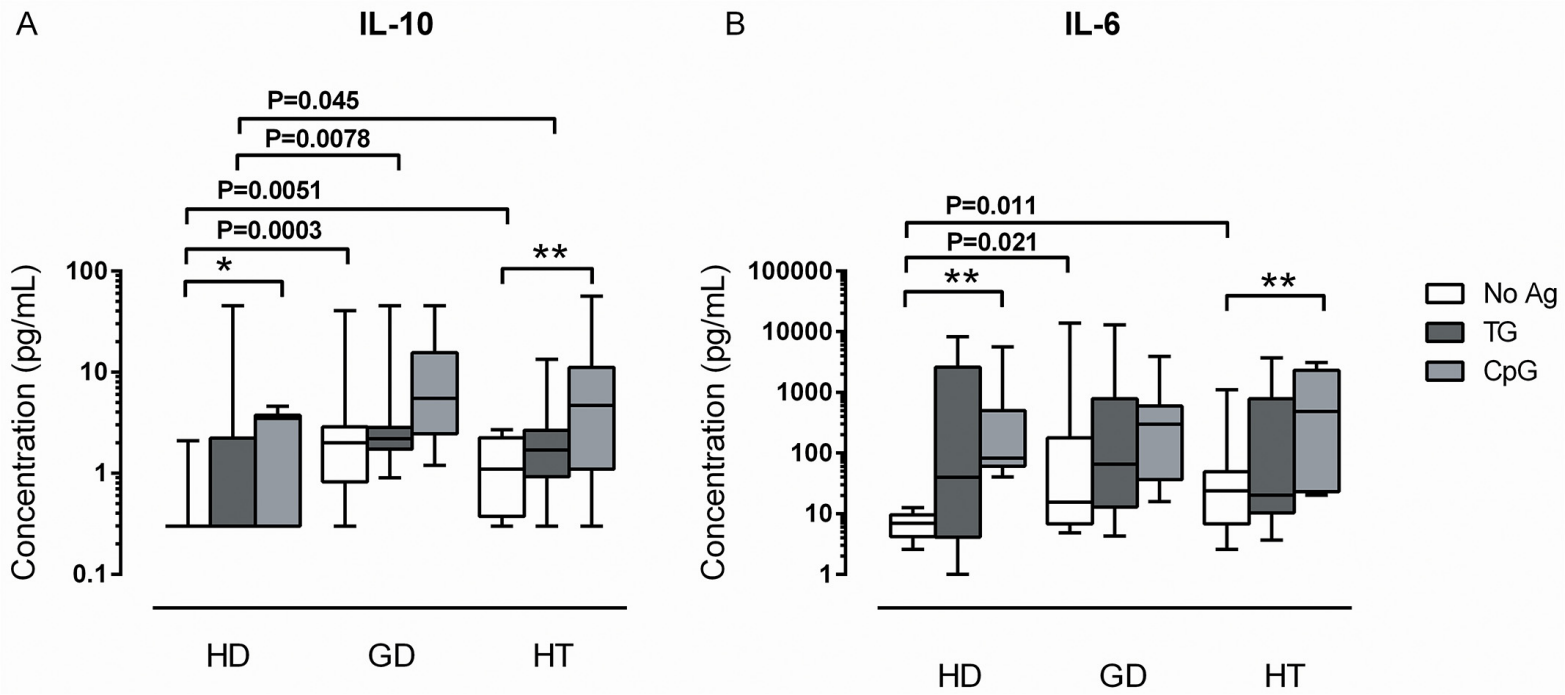
Secretion of IL-10 and IL-6 in co-cultures of TG-stimulated purified B cells and the remaining PBMCs. B cells from healthy donors (HD; N = 10) and patients with Graves’ disease (GD; N = 12) or Hashimoto’s thyroiditis (HT; N = 12) were pulsed with thyroglobulin (TG) and added back to the remaining PBMCs as described in Fig 1. After 48 h, the (A) IL-10 and (B) IL-6 secreted into culture supernatants were measured using the Luminex platform. The box plots indicate median, interquartile range (box) and range (whiskers). Brackets show differences between groups with the corresponding raw P-values (Mann-Whitney U test). * P<0.05; ** P<0.01 within groups (Wilcoxon matched-pairs signed rank test).

As a supplement to the measurement of intracellular IL-10, levels of IL-10 released into the culture supernatants was determined. Unexpectedly, greater IL-10 production was observed in both patient groups compared with healthy donors in the presence of B cells not pulsed with TG (Fig 2A). The same applied to the production of the pro-inflammatory cytokine IL-6, the levels of which were one to two orders of magnitude higher than those of IL-10 (Fig 2B). Pulsing of B cells with TG did not significantly alter these cytokine profiles. Therefore, baseline secretion of IL-10 and IL-6 by un-stimulated PBMCs from AITD patients is higher than in healthy donors independent of addition of exogenous self-antigen.

### IL-10^+^ B-cell proportions in AITD patients and healthy donors

**Fig 3:**
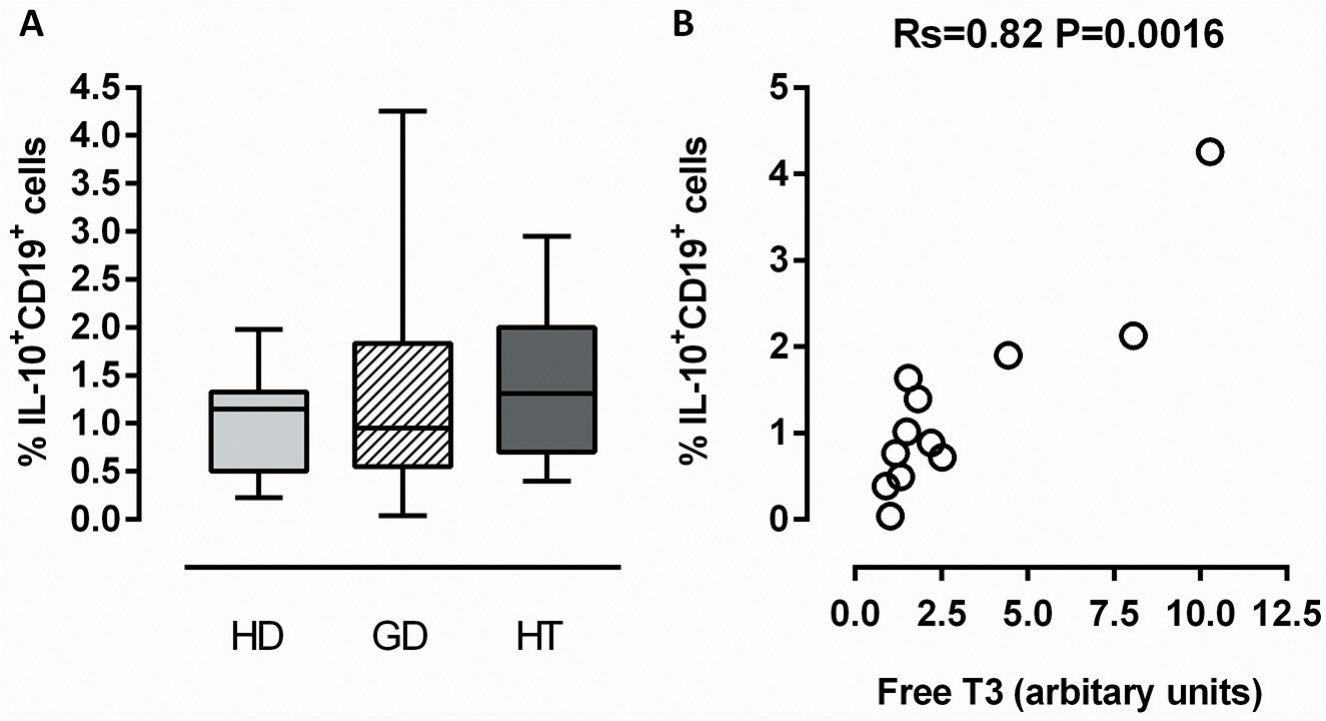
Maximal induction of IL-10^+^ B cells in healthy donors and AITD patients. PBMCs from healthy donors (HD; N = 12; light grey bars), patients with Graves’ disease (GD; N = 12; hatched bars) or patients with Hashimoto’s thyroiditis (HT; N = 12; dark grey bars) were stimulated with PMA/ionomycin for 4 h, and CD19^**+**^ B cells were analyzed for intracellular IL-10 content. (A) The percentages of IL-10^**+**^CD19^**+**^ B cells after stimulation are presented for each donor category. Box plots indicate median, interquartile range (box), and range (whiskers). (B) Correlation between PMA/ionomycin-induced IL-10^**+**^CD19^**+**^ B cell numbers and circulating FT_3_ levels in GD patients is shown. All values in have been adjusted for background frequency of positive events (after no stimulation).

To compare the maximal achievable IL-10^+^ B-cell production in the two patient groups with those of healthy controls, polyclonal stimulation of PBMCs with PMA/ionomycin was used to induce IL-10 expression (Fig 3A). Approximately, 1% IL-10^+^ B cells were identified following this stimulation in all three donor categories (Fig 3A).

A significant positive correlation was found between FT_3_ levels and the frequency of IL-10^+^ B cells in GD patients (P = 0.0016, Fig 3B) while a borderline-significant correlation was identified between FT_4_ levels and IL-10^+^ B-cell frequency (P = 0.059; data not shown). However, no correlation was found between IL-10^+^ B-cell abundance and serum TSH (data not shown).

The total B-cell count did not differ between healthy donors and patients with GD or HT (data not shown).

### Surface marker expression by IL-10^+^ B cells and IL-10^-^ B cells

B cells from healthy donors and patients with GD or HT were assessed for the phenotypes associated with regulatory functions. These include those cells displaying CD5, CD25, TIM-1, and combinations of CD24, CD27, CD38, and CD43 [12, 16–24].

**Fig 4:**
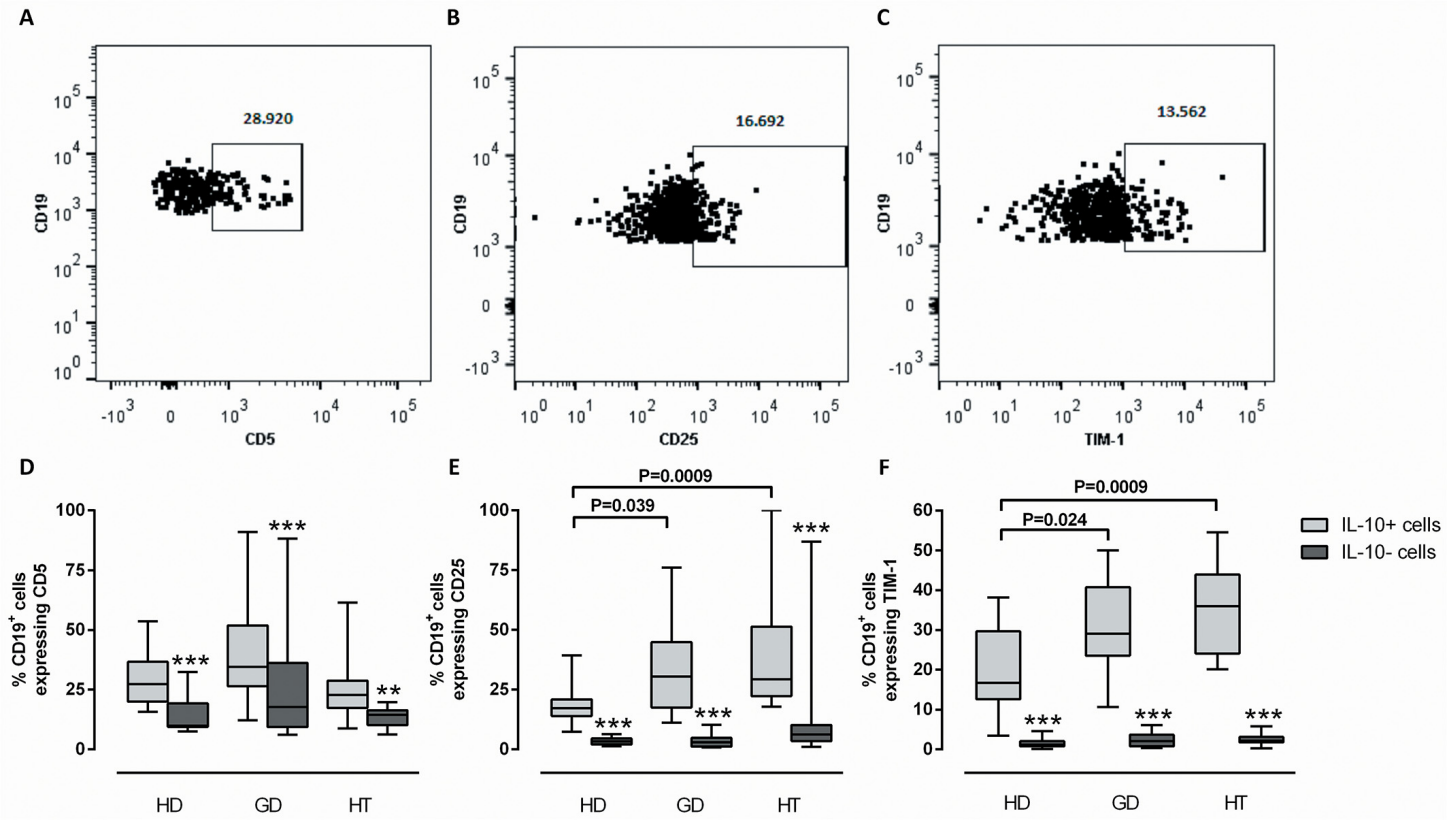
Frequencies of IL-10^+^ B cells expressing CD5, CD25 and TIM-1. PBMCs from healthy donors (HD; N = 12), patients with Graves’ disease (GD; N = 12) or patients with Hashimoto’s thyroiditis (HT; N = 12) were stimulated with PMA/ionomycin and stained with antibodies against CD19, IL-10, CD5, CD25, and TIM-1. The proportion of (A) CD5^**+**^, (B) CD25^**+**^ and (C) TIM-1^**+**^ cells within the IL-10^**+**^CD19^**+**^ B cell subset was analyzed. The frequencies of IL-10^**+**^ B cells (light grey) and the remaining B cells (IL-10^**-**^; dark grey) expressing (D) CD5, (E) CD25 and (F) TIM-1 are shown for each donor category. The box plots indicate median, interquartile range (box) and range (whiskers). Brackets show differences between groups with the corresponding raw P-values (Mann-Whitney U test). ** P<0.01; *** P<0.001 between IL-10^**+**^ cells and IL-10^**-**^ B cells within each group (Wilcoxon matched-pairs signed rank test).

Representative dot plots of CD5, CD25 and TIM-1 surface expression on IL-10^+^ B cells from a healthy donor are shown in Fig 4A–4C. As expected from earlier studies, IL-10^+^ B cells were enriched with all three markers, compared to the rest of the B-cell pool, in all three donor groups (Fig 4D–4F). The median proportion of CD25^+^IL-10^+^ B cells constituted 30% in both GD and HT, compared to only 17% in healthy donors (P = 0.039 and P = 0.0009, respectively; Fig 4E).

IL-10^+^TIM-1^+^ B cells were more abundant in GD and HT than IL-10^+^ TIM-1^+^ B cells from healthy donors (P = 0.024 and P = 0.0009, respectively; Fig 4F). These data should be interpreted with caution; however, since the 4 h PMA/ionomycin stimulation used to induce differentiation of IL-10^+^ B cell more than doubled the frequency of B cells displaying TIM-1 (S1 Table).

**Fig 5:**
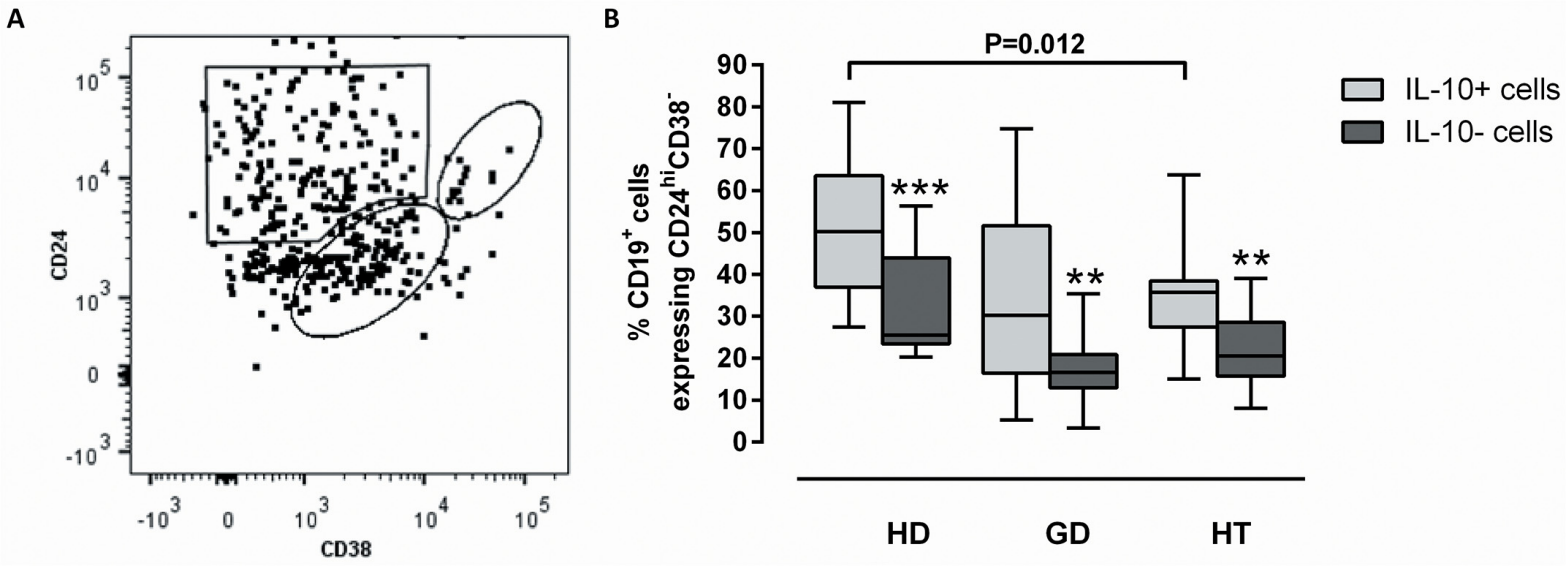
Frequencies of IL-10^+^ B cells expressing combinations of CD24 and CD38. PBMCs from healthy donors (HD; N = 12), patients with Graves’ disease (GD; N = 12) or patients with Hashimoto’s thyroiditis (HT; N = 12) were stimulated with PMA/ionomycin for 4 hours, stained with antibodies against CD19, IL-10, CD24, and CD38, and analyzed by flow cytometry. (A) The gating for three combinations of CD24 and CD38 was performed on IL-10^**+**^CD19^**+**^ cells from a healthy donor, representative of all three donor groups. (B) The frequencies of IL-10^**+**^ B cells (light grey) and the remaining B cells (IL-10^**-**^; dark grey) being CD24^**hi**^CD38^**-**^ are shown for each donor group. The box plots indicate median, interquartile range (box) and range (whiskers). Brackets show differences between groups with the corresponding raw P-values (Mann-Whitney U test). ** P<0.01; *** P<0.001 between IL-10^**+**^ cells and IL-10^**-**^ B cells within each group (Wilcoxon matched-pairs signed rank test).

The CD24^hi^CD38^hi^ and CD27^+^CD43^+^ phenotypes, previously associated with Breg function [22, 23, 25], were also investigated. Only a minority of IL-10^+^ B cells were CD24^hi^CD38^hi^ (Fig 5A), regardless of the donor group, and the proportion of these cells was similar in all three donor groups (data not shown). IL-10^+^ B cells were predominantly found within the CD24^hi^CD38^-^ memory B-cell compartment in healthy donors (Fig 5B), while they were underrepresented in this compartment in HT patients (p = 0.012; Fig 5B). HT patients had a correspondingly higher proportion of mature CD24^int^CD38^int^IL-10^+^ B cells than healthy donors (37% vs. 21%, respectively, p = 0.0023; data not shown).

The median proportion of IL-10^+^ B cells expressing the CD27^+^CD43^+^ phenotype comprised 19% in healthy donors and 29% in both patient groups (NS; data not shown).

## Discussion

In this study, we examined the induction of IL-10^+^ B cells by the thyroid self-antigen TG, and by the polyclonal stimulators PMA/ionomycin and CpG. We consider TG to represent a more physiologically relevant stimulus than the mitogenic stimuli normally used to study regulatory B cells. In keeping with our previous findings in healthy donors [12], TG induced significant IL-10^+^ B-cell differentiation in GD, and a similar tendency was observed in healthy donors. These data suggest that IL-10^+^ B-cell differentiation in GD is not compromised. It should be noted, however, that we did not assess TSH receptor-reactive IL-10^+^ B cells, which are presumably more relevant to the pathogenesis of GD. Interestingly, B cells pulsed with TG were capable of inducing IL-10-producing CD4^+^ T cells after being added back to PBMCs cultures from GD patients. This is in keeping with our previous findings in cells from healthy donors [12].

No statistically significant changes in IL-10 or IL-6 levels could be detected in culture supernatants following exposure of B cells to TG, but trends confirmed our previous findings that B cells pulsed with TG can induce production of both cytokines – as well as tumor necrosis factor (TNF)-α and TGF-β – in co-cultures with autologous T cells [12]. While the three donor groups did not differ with respect to the ability of TG to induce cytokine responses, the baseline production of both IL-10 and IL-6 was greater in the two patient groups. Considerably more IL-6 than IL-10 was produced, which might reflect a more pro-inflammatory environment in AITD patients compared to healthy donors. We have previously shown that addition of TG to PBMCs induced increased production of TNF-α, IL-2, interferon-γ and IL-10 in GD patients and HT patients, compared to healthy donors [26]; the data presented here suggest that B cells pulsed with TG do not provide a stimulus strong enough for similar changes in cytokine production.

We observed no difference between healthy donors and either patient group with respect to induction of IL-10^+^ B cells by the polyclonal stimuli PMA/ionomycin or CpG. In contrast to our results, Zha *et al*., also using CpG and PMA/ionomycin as stimuli, observed a significantly lower frequency of IL-10 producing B cells in new-onset GD patients than in healthy donors [27]. The reason for this discrepancy may be that Zha *et al*., investigated new-onset GD patients, while those with GD in our study cohort had disease of a longer duration, and 9 out of 12 of them had received methimazole. Significantly decreased IL-10^+^ B-cell frequency has also been described in patients with rheumatoid arthritis [28, 29]. Importantly, our finding of similar frequencies of IL-10^+^ B cells in healthy donors and AITD patients does not rule out disease-associated B cell impairment with respect to IL-10-independent regulatory mechanisms affecting T-cell activity [27]. Alternative ways for B cells to regulate T-cell functions include mechanisms mediated by TGF-β [30], and expression of surface molecules such as programmed death ligand 2 [9, 31] and Fas ligand [9, 32, 33].

Unexpectedly, the frequency of induced IL-10^+^ B cells correlated positively with FT_3_ and FT_4_ levels (the latter borderline significance), within the GD patients. The increase in IL-10^+^ B cell numbers may reflect an attempt of the B cell compartment to limit or eliminate disease activity. This has been demonstrated in experimental autoimmune encephalomyelitis where wild-type mice spontaneously remit or even recover within 30 days, but mice with a selective lack of IL-10 expression in B cells fail to do so [7]. Recently, we showed that the frequency of IL-10^+^ T cells is inversely correlated with TSHR-antibody levels, a marker of disease activity, in GD patients [34]. The increasing IL-10^+^ B-cell frequencies with increasing FT_3_ levels reported in this study may reflect compensation for the relative IL-10^+^ T cell deficiency.

Within the IL-10^+^ B cell subset, a greater proportion of cells displaying CD25 or TIM-1 were found in patients with HT or GD than in healthy donors. CD25 is the IL-2 receptor α-chain, which promotes B-cell and T-cell proliferation [35]. Expression of CD25 may allow IL-10^+^ B cells to become activated – and regulate the immune response – under circumstances with abundance of IL-2 in the environment, i.e. in presence of activated effector T cells. Our data confirm that IL-10^+^ B cells are enriched with CD25^+^ cells, as compared with the entire B cell population, but 50–75% of IL-10^+^ B cells did not express this marker. TIM-1 is a T-cell co-stimulatory molecule which regulates CD4^+^ T-effector cell differentiation and responses in autoimmune and alloimmune settings [21, 36, 37]. Ligation of TIM-1 induces IL-10 production by TIM-1^+^ B cells and, in so doing, may promote immune tolerance [21]. The finding of a significantly increased proportion of TIM-1^+^IL-10^+^ B cells in HT and GD may thus reflect a compensatory increase in Bregs in this group. The findings concerning TIM-1 in this study should be interpreted with caution since TIM-1 expression on the B-cell surface was greatly enhanced by PMA/ionomycin stimulation. Even so, the majority of IL-10^+^ B cells in each group lacked this marker.

Immunoregulatory properties have been associated with CD24^hi^CD38^hi^ B cells, a phenotype which defines transitional B cells [22, 23, 28, 38], and which has been reported to be underrepresented in active RA [28]. In our study, the proportions of CD24^hi^CD38^hi^ B cells were similar between healthy donors and patients with GD or HT; however, IL-10^+^ B cells were predominantly CD24^hi^CD38^-^ (memory) cells in healthy donors, but almost evenly distributed among CD24^hi^CD38^-^ (memory) cells and CD24^int^CD38^int^ (mature) cells in both patient groups. Others have also found IL-10 production within the CD24^int^CD38^int^ and CD24^hi^CD38^-^ phenotypes, indicating that these subsets may also exert immunosuppressive activity [23]. We found no difference between the donor groups with respect to the frequencies of IL-10^+^ B cells with the CD27^+^CD43^+^ phenotype, which has also been associated with IL-10 production and immunosuppression [24, 25]. We did not examine CD24 in combination with CD27, but Zha *et al*., found a lower frequency and functional impairment of the CD24^hi^CD27^+^ B cells in recent onset GD [27].

Among the subjects in this study, 9 out of 12 GD patients were undergoing anti-thyroid drug treatment, and 5 out of 12 HT patients were receiving levothyroxine treatment at the time of blood collection. It has been reported that anti-thyroid drugs may inhibit lymphocyte function and reduce production of pro-inflammatory cytokines, in part by inhibiting NF-κB [1, 39–41]. Due to the potentially normalizing effects of anti-thyroid treatment, differences between patients and healthy donors with respect to IL-10 production may have been underestimated in this study. It should also be noted that IL-10 production by circulating B cells might not mirror production in intra-thyroidal B cells.

In conclusion, differentiation of B cells into IL-10-producing cells was unimpaired in GD and HT after stimulation with TG as well as with PMA/ionomycin. As has been observed previously, IL-10^+^ B cells did not segregate into clearly defined phenotypic subsets, regardless of the donor group examined. Future treatment of AITD should be aimed at reinstituting immune tolerance in these patients with antigen-specific therapies. Our observation that pulsing of B cells with TG induces IL-10^+^ T cells as well as IL-10^+^ B cells in GD patients suggests that loading of B cells with appropriate self-antigens may be exploited in this respect.

## Supporting Information

S1 Table Expression of surface markers by bulk B cells before and after PMA/ionomycin stimulation. PBMCs from healthy donors (N = 6) were stimulated with phorbol 12-myristate 13-acetate/ionomycin (PMA/ionomycin) or left unstimulated (baseline) for 4 hours. The proportion of B cells expressing each surface marker after stimulation was related to that of unstimulated B cells as a ratio. The median value of the ratios of 6 individual donors is shown.
(DOCX)

S1 Fig Gating strategy defining IL-10^+^ B cells. Representative dot plots show unstimulated CD19^+^ B cells (left panel) versus PMA/ionomycin-stimulated CD19^+^ B cells (right panel). The gate defining IL-10^+^ B cells was used throughout the paper.
(TIF)
