# Supplementary material for: Characterization of Regulatory B Cells in Graves’ Disease and Hashimoto’s Thyroiditis
Source: PLoS One. 2015 May 27;10(5):e0127949. doi: 10.1371/journal.pone.0127949 (PMC4446335; doi:10.1371/journal.pone.0127949)
Supplement: S1 Table — PBMCs from healthy donors (N = 6) were stimulated with phorbol 12-myristate 13-acetate/ionomycin (PMA/ionomycin) or left unstimulated (baseline) for 4 hours. The proportion of B cells expressing each surface marker after stimulation was related to that of unstimulated B cells as a ratio. The median value of the ratios of 6 individual donors is shown. (DOCX) [file pone.0127949.s001.docx]

|  | Without PMA/iono | With  PMA/iono | Ratio  without: with  PMA/iono |
| --- | --- | --- | --- |
| CD5 | 5.5 % | 5.1 % | 0.96 |
| CD27 | 30.8 % | 24.5 % | 0.80 |
| CD43 | 2.2 % | 2.8 % | 1.05 |
| CD25 | 8.7 % | 6.9 % | 0.64 |
| TIM-1 | 0.4 % | 0.9 % | 2.37 |
| CD24hi | 40.3 % | 41.0 % | 1.02 |
| CD24int | 57.7 % | 57.8 % | 1.01 |
| CD38hi | 4.6 % | 4.9 % | 1.05 |
| CD38int | 61.3 % | 54.9 % | 0.94 |
|  |  |  |  |

**S1 Table**. Expression of surface markers by bulk B cells before and after PMA/ionomycin stimulation.

Peripheral blood mononuclear cells (PBMCs) from healthy donors (N=6) were stimulated with phorbol 12-myristate 13-acetate/ionomycin (PMA/iono) or left unstimulated (baseline) for 4 hours. The proportion of B cells expressing each surface marker after stimulation was related to that of unstimulated B cells as a ratio. The median value of the ratios of 6 individual donors is shown.
