# Supplementary figures and images for: Characterization of Regulatory B Cells in Graves’ Disease and Hashimoto’s Thyroiditis
Source: PLoS One. 2015 May 27;10(5):e0127949. doi: 10.1371/journal.pone.0127949 (PMC4446335; doi:10.1371/journal.pone.0127949)

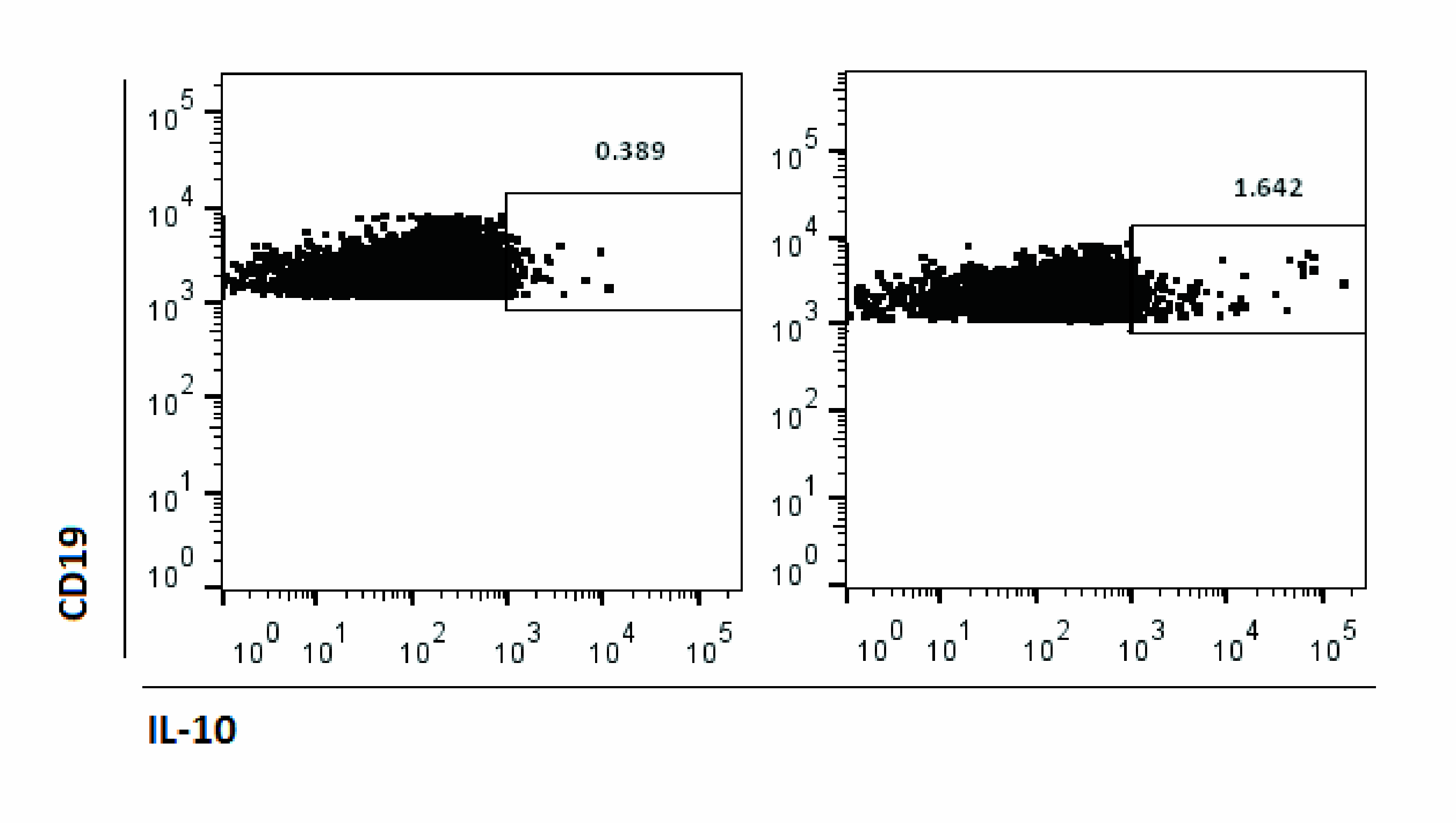

Supplement: S1 Fig — Representative dot plots show unstimulated CD19+ B cells (left panel) versus PMA/ionomycin-stimulated CD19+ B cells (right panel). The gate defining IL-10+ B cells was used throughout the paper. (TIF) [file pone.0127949.s002.tif]
